# Bioactive Composite Membranes: Preclinical Analysis of PBAT/BAGNb Composition to Enhance the Quality of Guided Bone Regeneration

**DOI:** 10.1021/acsami.4c20580

**Published:** 2025-02-12

**Authors:** Gabriela de Souza Balbinot, Vicente Castelo Branco Leitune, Rosane Michele Duarte Soares, Fernanda Visioli, Deise Ponzoni, Fabricio Mezzomo Collares

**Affiliations:** † Dental Materials Laboratory, School of Dentistry, Universidade Federal do Rio Grande do Sul, 90035-003 Porto Alegre, RS, Brazil; ‡ Polymeric Biomaterials Laboratory (Poli-BIO), Institute of Chemistry, Universidade Federal do Rio Grande do Sul, 91501-970 Porto Alegre, RS, Brazil; § Pathology Laboratory, School of Dentistry, Universidade Federal do Rio Grande do Sul, 90035-003 Porto Alegre, RS, Brazil; ∥ Oral and Maxillofacial Surgery Unit, Universidade Federal do Rio Grande do Sul, 90035-003 Porto Alegre, RS, Brazil

**Keywords:** regenerative medicine, tissue engineering, guided tissue regeneration, animal model, biodegradable plastics, niobium

## Abstract

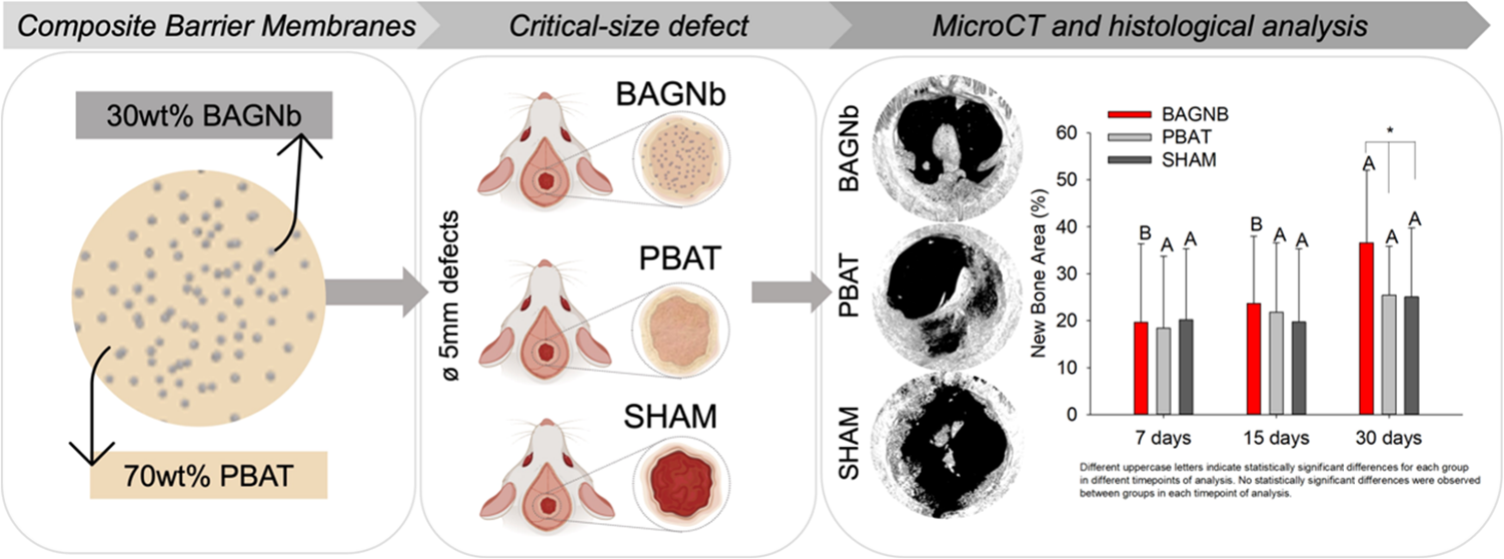

Bioactive barrier membranes aim to actively contribute to bone formation by promoting cell proliferation, differentiation, and mineral deposition into bone defects. Unlike traditional membranes, they can actively promote bone growth by releasing ions, fostering an optimal environment for GBR. In this study, we aimed to investigate the preclinical behavior of PBAT/BAGNb membranes in a critical-sized defect model in rat calvaria. A flexible and resorbable polyester (poly(butylene adipate-*co*-terephthalate)) (PBAT) was combined with 30 wt % of niobium-containing bioactive glasses (BAGNb) to produce bioactive composites that were used as membranes for repairing critical-sized calvaria bone defects (Ø5 mm) in an early-stage regeneration preclinical model. The bone formation was evaluated via X-ray microtomography and histological analysis. X-ray microtomography measurements revealed enhanced bone formation in the group treated with the bioactive composite membranes (PBAT/BAGNb) compared to those treated with pure PBAT membranes or left empty (SHAM). The morphometric analysis demonstrated a more densely packed trabecular structure in the newly formed bone of the BAGNb group, indicating tissue maturation within the defects. Histological sections showed minimal signs of inflammation associated with PBAT-based membranes, and mature bone tissue gradually formed with BAGNb in the membranes over time. The preclinical evaluation of PBAT/BAGNb demonstrated enhanced mineral formation and a well-organized trabecular structure, indicating successful outcomes for their use in GBR procedures. A PBAT/BAGNb composite combined flexibility and bioactivity to enhance early bone regeneration, improving mineral formation and a more organized bone structure.

## 1 Introduction

Osseointegration has revolutionized the biomedical field creating favorable conditions for implant rehabilitations, marking a milestone in improving the quality of life for people requiring dental and orthopedic rehabilitation.^1^ It is estimated that around 50% of dental implant placements require some bone augmentation, which leads to a global annual expenditure of more than US$720 million. Guided bone regeneration (GBR) is a technique that supports bone augmentation by maintaining and restoring tissue quality and quantity for implant-supported rehabilitation.^2−5^ Key factors in its success include maintaining space for bone growth and protecting the surgical site. This prevents epithelial tissue invagination, allowing osteogenesis through bone cell migration from native tissue.^6−8^ Based on the known principles of guided bone regeneration, currently used membranes are understood to be inert, lacking active contributions to new bone formation and tissue healing.^9^

Controlling the properties and activity in the barrier membranes could contribute to better surgical outcomes for GBR. Currently, commercially available membranes vary widely in properties. Besides being inert, they do not always fulfill the requirements for universal application in GBR procedures. For example, space maintenance is observed for metallic and poly(tetrafluoroethylene) (PTFE) membranes but they lack resorbability, increasing the number of surgical interventions and morbidity;^10−12^ collagen membranes, on the other hand, are resorbable structures with reduced mechanical properties and unstable occlusive capacity.^13−15^ Different organic and inorganic compounds have been studied to overcome the limitations of available membranes, combining resorbability and mechanical strength.^16−19^ A key component missing in these materials is, however, the ability to stimulate new bone formation with bioactive compounds.

Attempts to incorporate bioactive compounds into commercially available membranes have aimed to provide these materials with ion release capacity. Different barrier membrane formulations have been proposed to combine adequate mechanical properties for space maintenance, stable degradation for occlusive properties, and bioactive compounds to enhance tissue regeneration.^20^ Synthetic bioresorbable polyesters have been proposed for this aim due to their biocompatibility and the ability to tailor their properties for functional rehabilitation in GBR techniques.^21−23^ Poly(butylene adipate-*co*-terephthalate) (PBAT) has been recently described as a candidate for GBR membrane production due to its flexible aliphatic-aromatic polyester composition.^24^ This allows a low degree of crystallization for high flexibility and resorbability due to the aliphatic chains, while aromatic units are known to contribute to the material strength. The combination of these two units in a single polymer contributes to the handling of developed membranes, facilitating clinical application and yet maintaining the mechanical needed to protect the bone defect.^25^

Due to its flexibility, PBAT mechanical properties allowed the incorporation of a high concentration of bioactive ions as previously described.^17^ A previous report from our group evaluated the production of PBAT/BAGNb composites in vitro. The addition of 30 wt % of inorganic components led to suitable mechanical and surface properties allowing GBR principles, with BAGNB as a source of bioactive ions to improve the material’s ability to control cell–material interactions, promoting the proliferation and differentiation of osteogenic lineage cells. While *in vitro* findings suggest an effect of BAGNb ions and the suitability of the PBAT polymer as membrane material, an *in vivo* response is still required. Thus, the present study investigates the preclinical behavior of PBAT/BAGNb membranes in a critical-sized defect model in rat calvaria. The new bone formation was assessed via X-ray computed microtomography where tridimensional reconstructions were used to assess the amount and microstructure of newly formed bone over time.

## 2 Materials and Methods

### 2.1 Barrier Membrane Production

PBAT membranes were prepared as previously described.^17^ Poly(butylene adipate-*co*-terephthalate) (PBAT- Ecoflex F Blend C1200; BASF Corporation, Florham Park, NJ) pellets with 1.27 g/cm^3^ at 23 °C density were used. Hot-melt extrusion was used for the dispersion of bioactive glass particles into the PBAT using twin-screw extrusion (Haake H-25, Rheomex PTW 16/25-Polylabsystem, Karlsruhe, Germany) at 150 rpm rotation. Niobium-containing bioactive glasses were produced via the sol–gel^26^ and added to the PBAT before extrusion at 30 wt % concentration. Pure PBAT was extruded as well as a control. Solvent-casting was applied to the polymer and composites to produce membrane films with 0.20 mm thickness. Chloroform solution with a 1:7.5 (v/w) proportion was used to solubilize the materials under stirring for 24 h. The solution was then poured into a glass container at 37 °C for 1 h. Disk-shaped membranes measuring 6 mm in diameter and 0.20 mm in thickness were cut from the obtained film. Samples were sterilized in hydrogen peroxide before the surgical procedure. The characterization of these materials revealed membranes with no chemical interaction between the PBAT and BAGNb, evidenced by chemical compounds related to the presence of BAGNb, such as Si–O_2_ bonding, independent of the PBAT matrix. Also, BAGNb in this concentration promoted little impact on the thermal behavior of PBAT. When compared to the pure PBAT membranes, the 30 wt % addition of BAGNb resulted in a reduction in tensile strength and an increase in the elastic modulus in the composites, which is important for space-maintaining capacity in GBR membranes. The wettability was improved with the addition of BAGNb. The BAGNb at 30 wt % concentration into PBAT promoted enhanced cell viability and modified the mineralization response in vitro, showing an increased ability to mineralize for osteoblastic cells treated with these membranes when compared to pure PBAT.^17^

### 2.2 Study Design

**Figure 1:**
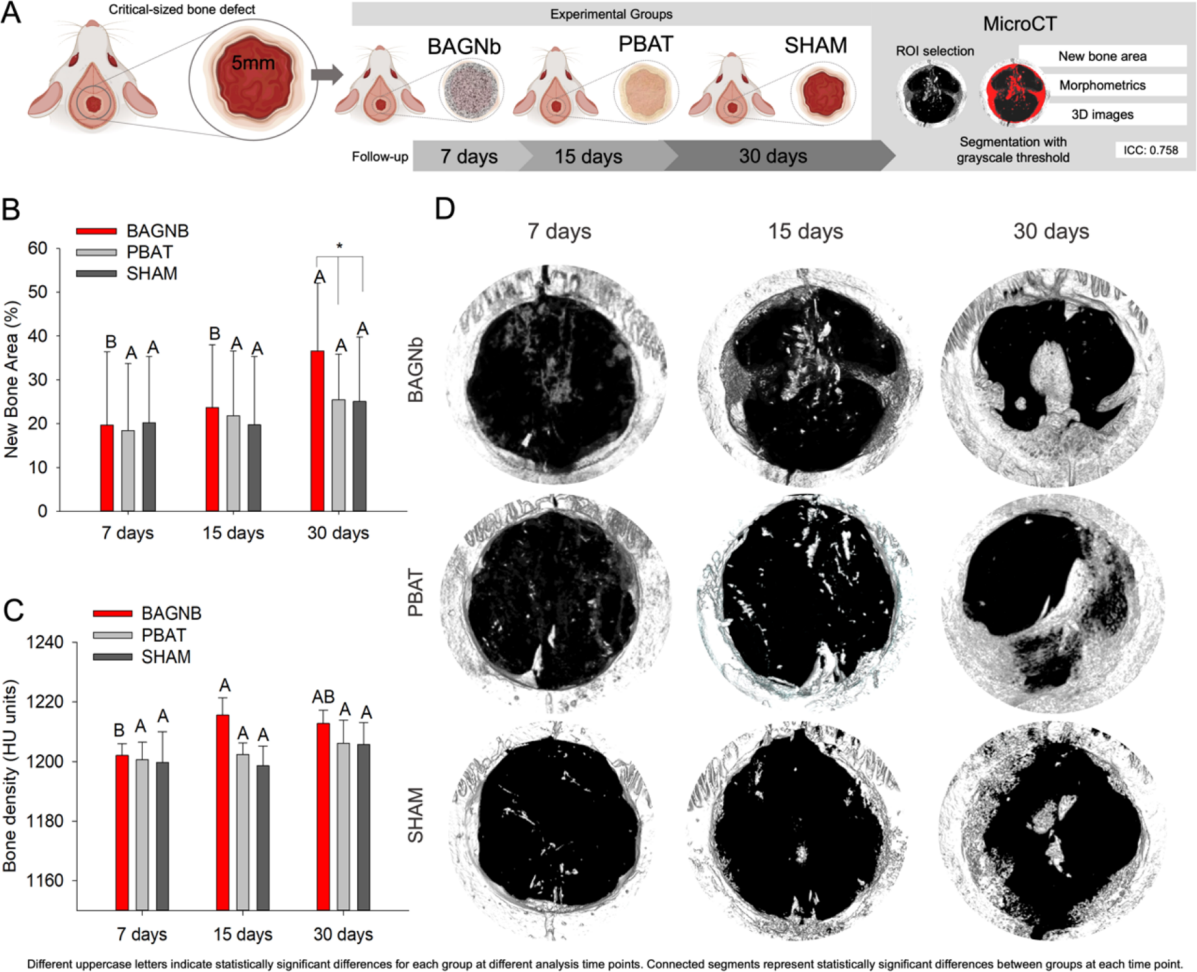
Percentage of new bone formation into the critical-sized defects (*n* = 7). (A) Schematics representing the defect production and study design with microCT segmentation for bone quantification. (B) Average values for new bone formation and (C) bone density. (D) Representative 3D images from different groups and different time points of analysis. Different uppercase letters indicate statistically significant differences for each group at different time points of the analysis. Connected segments represent statistically significant differences between groups at each time point.

Sixty-three (*n* = 7) male rats (*Rattus novegicus albinus*, *Rodentia mammalia*—Wistar lineage) with an average weight of 500 g were used. The animals were maintained in appropriate cages in a controlled temperature range (20–24 °C) and light/dark cycle with food and water provided *ad libitum*. Environmental enrichment was used in the cages. All procedures were performed according to the Guide for the Care and Use of Laboratory Animals and with approval from the local ethical committee (Porto Alegre University Hospital – HCPA #2020-0075). ARRIVE guidelines were followed for the report of experimental procedures (Figure S1). The animals were weighed and randomized by a blinded veterinarian using random sequence generation, into their cages, according to the different treatments to ensure similar weight in all groups. Three experimental groups were designed: animals assigned to the BAGNb group received PBAT membranes with 30 wt % bioactive glass incorporation; the pure PBAT membranes were used for the PBAT group; as a control group, empty defects were used in the SHAM group (Figure 1A).

### 2.3 Surgical Procedure

The surgical procedure was performed after anesthesia with an intraperitoneal administration of ketamine (50 mg/kg), xylazine (5 mg/kg), and 1–2% vaporized isoflurane for maintenance. The calvaria was assessed after shaving, skin antisepsis with 2% chlorhexidine, and local anesthesia with bupivacaine 5%/adrenaline 1:200,000. A 5-cm-long incision was made, and the calvaria bone was exposed. A critical defect was created in the center of the calvaria bone with a Ø5 mm trephine burr using a low-speed drill. The trephine was positioned caudal to the bregma, and the defect was produced with constant irrigation until a thin section of bone was observed. To prevent dura mater injuries, a Ø1 mm carbide bur was used in the final portion of the bone. The calvaria bone tissue was then delicately separated from the dura mater with a spatula. Bleeding was controlled before intervention, and in case excessive bleeding was detected, the animals were excluded from the sample to avoid bias in the analysis. The researcher responsible for the surgical procedure was informed of the allocation of animals according to the randomization only after the defect was completely produced. For BAGNb and PBAT groups, the membranes were gently positioned on top of the defect before the suture of periosteal flaps using a 4.0 glycolide/l-lactide copolymer (Figure 1A). The skin was then sutured with 4.0 wt % nylon. Postoperative analgesia was performed immediately with a single dose of morphine (5 mg/kg) administered via intraperitoneal injection and tramadol (20 mg/kg) for 5 days in a 12/12 h cycle. The animals were observed for signs of pain and possibly neurological disorders. After 7, 15, and 30 postoperative days, the animals were euthanized with an isoflurane overdose. The calvaria tissue was then removed by drilling, and samples were immediately immersed in 10% formalin solution before their characterization.

### 2.4 X-ray Computed Microtomography

Calvaria defects (*n* = 7) were evaluated by X-ray computed microtomography (MicroCT.SMX-90 CT; Shimadzu Corp., Kyoto, Japan). Before being scanned, the samples were gently washed with distilled water for 30 s. Then, the samples were imaged with 360° rotation, 60 kV voltage, and 110 mA current with a 1 mm AL filter. The resultant mages were obtained with 1024-pixel resolution and a 12 μm voxel size. Tridimensional reconstruction was performed with DICOM files in image software (RadiAnt DICOM Viewer, Medixant, Poznan, Poland). Measurements were performed with a 2D image stack. For measuring new bone area and bone density, a standardized area (5 mm^2^) was selected as the region of interest (ROI) for the measurements and a 2.0 Gaussian blur filter was used for smoothing. Thresholding (100–255) was applied in the binary images to segment new bone tissue, and the thresholding was the same for all groups, to ensure that only bone tissue was selected, and porous areas were found were not considered in the measurement (Figure 1A). The percentage of the new bone area was assessed as the primary outcome and the values obtained in each image were used to observe differences in the new bone area in de defect extent. Additionally, morphometric measurements were assessed by using the BoneJ plugin^27^ (ImageJ; National Institutes of Health, Bethesda, MD). The bone volume fraction measurements (BV/BT), bone connectivity (ConnD), trabecular thickness (Tb.Th), trabecular separation (Tb.Sp), and trabecular number (Tb.N) were measured to assess the percentage covered defect and how trabecular structure was formed for each intervention. The examiner that performed the analysis was blinded to the conditions and calibrated before measurements via an interclass correlation coefficient (ICC) test applied after training to assess the concordance in the measurements.

### 2.5 Histological Analysis

Histological analysis was performed after sample decalcification in EDTA 12.5% (v/v) for 4 weeks with daily solution exchange to remove the mineralized portion of bone and observe the bone matrix and modifications in bone repair at the cellular level. After decalcification, tissue was included in paraffin blocks and the block was sectioned in the sagittal direction. Then 4 μm-thick sections were produced. Staining with hematoxylin and eosin following washing and staining protocol.^28^ Slices were then imaged under 40× magnification (QCapture software, QImaging, British Columbia, Canada) for descriptive and quantitative analysis. The description was performed based on the presence of inflammatory infiltrate, fibrous capsule formation, and the maturation of newly formed bone. For quantitative analysis, the new bone formation was assessed in the stained slices based on segmentation based on trainable image processing (ImageJ; National Institutes of Health, Bethesda, MD). This tool allows machine learning-based segmentation considering the differences in pixel colors and it was used to separate the connective tissue, the bone area, and the background in each image, as exemplified in Figure 3A. New bone formation was evaluated at a standardized area of 4 mm^2^ in the central portion of the defect. The percentage of area occupied by bone was calculated by a blinded researcher trained in the tool, and measurements were performed after calibration via interclass coefficient correlation (ICC).

### 2.6 Statistical Analysis

The sample size was calculated based on previous studies^29,30^ considering the minimum detectable difference in means of the observed effect size for new bone formation measurements as the primary outcome with a 20% increase to account for possible loss in the sample. The interclass correlation coefficient (ICC) was used for calibration in microCT and histological measurements. Examiners measured a set of 10 random images in the sample in triplicate, and these values were used to detect reliability in a random effects model. The ICC analysis conducted with the percentage of new bone formation values from microCT resulted in 0.758, while for quantification in histological slices, it was 0.869. In both cases, the reliability was considered excellent (0.75–1.00. *p* < 0.05). 3D images and histology images were descriptively analyzed. The normality of the data was assessed using the Shapiro–Wilk test. Comparisons among different groups and postoperative times were performed with two-way ANOVA followed by the Tukey *post hoc* test. All tests were performed at a 5% significance.

## 3 Results and Discussion

Barrier membranes are a key component for bone augmentations with guided bone regeneration,^6^ and the currently available materials have limitations in handling and bioactive properties. While their role in new bone formation is influenced by their ability to modulate tissue reaction,^31^ the commercially available products lack any interaction with host tissues. Bioactive membranes produced with PBAT/BAGNb composites were able to be applied to critical-size defects and enhance new bone formation, creating conditions for the principles of GBR along with faster and more effective ossification. The preclinical application was successful. After 30 days, the BAGNb group exhibited increased bone area and volume with a more organized trabecular structure. These findings suggest that the proposed composition enhances the tissue response, supporting its potential use in guided bone regeneration.

This is the first preclinical study that describes the use of PBAT as a membrane for the barrier in GBR. A previous in vitro analysis reports the characterization of PBAT/BAGNb membranes, showing that PBAT is a good candidate for GBR membrane production, while BAGNb could be used to enhance the biological properties while maintaining the requirements for GBR.^17^ The application of this composition as a resorbable polyester for barrier membranes was successful considering the requirements for this procedure: space maintenance, defect sealing, and preventing migration of cells from the epithelial tissue to the bone.^8^ These principles are pivotal for a membrane in the GBR concept, requiring the barrier material to maintain stability throughout tissue regeneration.^20^ To ensure the safety of applied procedures and materials, we monitored 60 animals post-surgery, observing no complications or signs of pain and suffering. Following the ethics guidelines (Figure S1), three animals were excluded from the sample during the surgical procedure due to unusual bleeding. Enhanced new bone formation with PBAT-based membrane implantation, compared to the SHAM group, highlights these materials’ adherence to GBR principles (Figure 1). These findings show that PBAT, as a flexible polymer,^32,33^ has sufficient mechanical strength to prevent epithelial invagination and maintain space within the bone defect, effectively creating a barrier in the defect, while allowing flexible handling. PBAT composites containing 30 wt % BAGNb particles were easily applied to the bone defect as well. This composition presented increased stiffness and enhanced *in vitro*(17) and thus, a better space maintenance is expected, which may contribute to the findings of this study. Despite the differences in the mechanical properties, no postoperative complications were observed for the membrane groups.

In addition to the safety of the newly developed materials, we observed differences in their effectiveness at promoting new bone formation among the tested groups. At 30 days, it is possible to observe a higher percentage of new bone for the BAGNb group (Figure 1B, *p* < 0.05). BAGNb group also presented higher new bone area at 30 days when compared to 7 and 15 days, which was not observed for PBAT and SHAM groups (Figure 1B). These results highlight the effectiveness of modifying the structure of PBAT to obtain bioactive composites for GBR. Considering the BAGNb composition, other *in vitro* and *in vivo* studies have investigated the role of niobium in bioactive glass compositions for bone regeneration,^34−36^ showing that the release of this ion may modulate tissue response by enhancing osteoblast differentiation into the defect site. In the present study, PBAT/BAGNb composites showed that 36.59% of the bone area was filled with new bone after 30 days, while for pure PBAT, new bone area reached 25.46%. The enhanced mechanical properties, the better wettability, and the ability to promote cell differentiation toward the mineralization in cell culture were shown for the 30 wt % BAGNb-containing membranes.^17^ These results may be related to the behavior in the PBAT/BAGNb group, confirming the ability of these materials to adequately interact with the native bone and stimulating new bone formation in the in vivo scenario.

While the differences in the mean values for new bone formation are our primary outcome, they do not fully explain the bone coverage in the defects. The distribution of newly formed tissue over the defect contributes to the understanding of new bone formation from the periphery to the defect’s central areas. The BAGNb group showed higher bone density at 15 days compared to other groups and relative to its own density at 7 and 30 days (Figure 1C). Representative 3D reconstruction (Figure 1C) shows that new bone is visible at 15 days, mainly in the periphery with bone growth in the central portions for BAGNb and PBAT at 30 days. Defects that were treated with BAGNb presented more new bone formation in the central areas (∼3 mm from the periphery), reaching the central portion of the defect and not only the defect margins, with a more homogeneous distribution. This may be related to the activation of osteogenic factors from the BAGNb structure.^8,37^ In bone regeneration, the primary stimuli for cell proliferation and differentiation arise from the native bone periphery, which is crucial for defect repair but limited to critical-size defects. The complementary stimuli from BAGNb in the membranes could contribute to the partial bridging observed for BAGNb due to cell–material interactions along with the bone defect in the early stages of bone regeneration (30 days) showing that bone deposition may be accelerated and increased using the proposed strategy (Figure 1). Similar values were observed for 30 days of analysis in the histological section (Figure 3C), confirming the microCT results. Also, this corroborates the hypothesis that besides the known effect of barrier membranes in tissue regeneration, the presence of inorganic bioactive particles may enhance bone formation.

**Figure 2:**
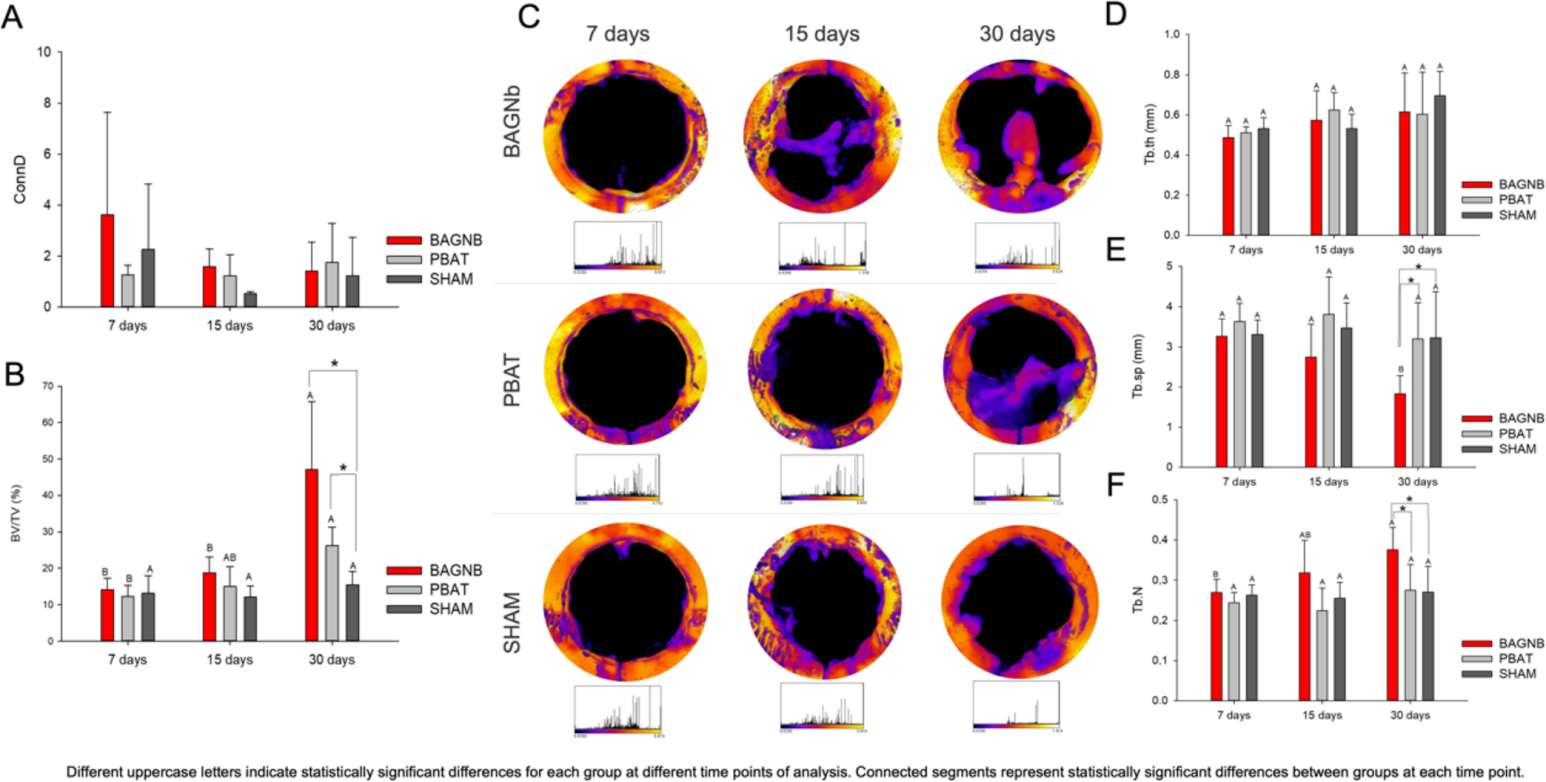
Morphometric structure in newly formed bone (*n* = 7). Connective density (ConnD; A) and the ratio between bone volume and total volume (BV/TV) in the analyzed samples (B) show that bone formation was increased by membrane application. (C) Representative reconstruction of sphere fitting for trabecular thickness measurements where purple regions are related to low values in trabecular thickness, while yellow/white regions represent higher values. Color histograms are shown below each image for scale. The average values for the images are shown in (D) where trabecular thickness (Tb.th) is presented; (E) trabecular separation (Tb.sp); and (F) trabecular number (Tb.N) shows a reduction in separation between trabecula and the number of trabecular connections in bone, respectively. The reduction in separation and increase in number indicate the maturation of tissue over time. Different uppercase letters indicate statistically significant differences for each group at different time points of analysis. Connected segments represent statistically significant differences between groups at each time point.

The presence of BAGNb also modified the bone microstructure into defects. This is observed in the morphometric measurements shown in Figure 2. An increase in bone volume for BAGNb and PBAT is observed when compared to that of the SHAM group at 30 days. No changes in bone volume were found for the SHAM group from 7 to 30 days (Figure 2B; *p* < 0.05). The trabecular parameters were used to assess the quality of new bone to predict the ability of function maintenance in the regenerated tissue.^38^ The trabecular thickness (Tb.th) was not modified either by the treatment or by the postoperative time (Figure 2D). Similar trabecular separation was observed for the tested groups at 7 and 15 days, while a reduction in values was observed for BAGNb at 30 days compared to PBAT and SHAM (Figure 2E). Modifications on the bone toward mature tissue are observed in orange/yellow, which is observed for BAGNb images in Figure 2C. After 30 days, an increase in the number of trabeculae (Tb.N) is found for BAGNb as well (Figure 2F). BAGNb is known to enhance mineral formation *in vitro* and *in vivo*, but new bone must be deposited and remodeled as native tissue. The initial time points showed low trabecular thickness and number (Figure 2D,F) with increased separation between trabecula (Figure 2E), meaning that little mineralized and immature tissue is being formed. Modifications in the bone microstructure were observed after 30 days with a reduction in the trabecular separation and an increase in both thickness and number of trabeculae for the BAGNb. These characteristics indicate a better organization in a more packed structure, observed usually in more mature tissue.

**Figure 3:**
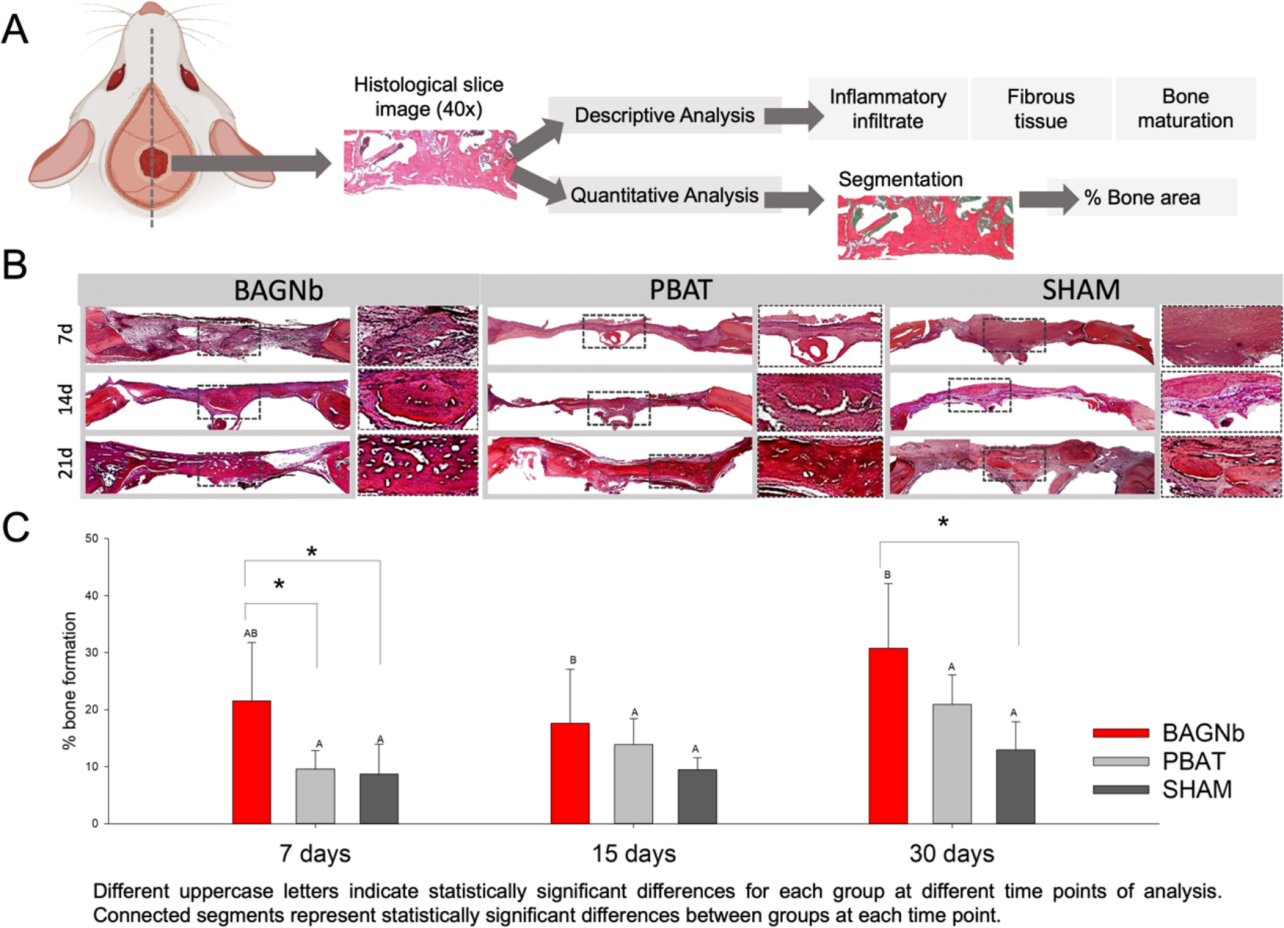
Histological analysis (*n* = 7). (A) Schematics of histological analysis with descriptive and quantitative analysis. (B) Representative images for each group and time point of H&E staining showing newly formed tissue in the whole defect (40x) with immature typical cellular content in low-mineralized bone at early time points, as evidenced in high magnification images. An increase in cell number denoting bone maturation is observed for BAGNb after 30 days. Minor inflammatory infiltrate was observed in the images. (C) Quantification of new bone into the defect based on threshold image to detect cellular components in the bone matrix in demineralized tissue. Different uppercase letters indicate statistically significant differences for each group at different time points of analysis. Connected segments represent statistically significant differences between groups at each time point.

These findings are supported by the histological analysis (Figure 3), where less organized bone structures are observed in the newly formed bone in the defect region (purple regions) showing, mostly, immature bone covering the bone defect at initial time points (Figure 3B). An increase in bone formation is observed over the defects with spare and highly cellularized trabecular structure at 30 days. Inflammatory cells are observed at 7 days and no evidence of exacerbated inflammation was detected. Increased bone formation was observed for the BAGNb group at 7 and 30 days when compared to SHAM (*p* < 0.05). For the BAGNb group, the fibrous structure at 15 days was substituted by a cellularized bone matrix, which indicates the bone deposition for bridging the critical-sized defect (Figure 3B). Bone regeneration strategies usually apply inorganic particles such as hydroxyapatite and other calcium phosphate structures that stimulate mineral formation without being properly resorbed to create functional bone tissue.^39^ The GBR concept itself usually combines membranes with bone graft filling for bone augmentations.^6,40^ When this combination of materials is tested, in similar animal models, new bone formation was comparable to the findings of the present study (Figure 1) and future studies may evaluate the application of these membranes alone or with a graft for direct comparison. While the use of particulate graft may be required depending on the surgical procedure needed, GBR may be applied in non-load-bearing conditions, being used alone with benefits in applying strategies such as the PBAT/BAGNb membranes.

Bioactive resorbable barrier membranes with tailored physical properties are a demand in the dental field to facilitate and enhance bone regeneration, allowing successful implant-supported rehabilitation. While initial results from microCT are shown in this study, further analysis with histological outcomes and biochemical markers will shed light on other aspects of new bone formation. The resorbability of PBAT into bone defects and the immune response promoted by either PBAT or BAGNb in the surgical sites may contribute to the understanding of the mechanisms behind the findings of the present study. Based on our results, the preclinical behavior of BAGNb-containing membranes indicated an increase in new bone formation with a more organized trabecular structure, more pronounced maturation of bone tissue, and minimal signs of inflammation in critical-size bone, which shows that the developed materials showed successful outcomes to be used in GBR procedures.

## 4 Conclusions

The preclinical evaluation of PBAT/BAGNb demonstrated enhanced mineral formation and a well-organized trabecular structure, indicating successful outcomes for their use in GBR procedures.

## Supporting Information Available

ARRIVE guidelines (Animal Research: Reporting of In Vivo Experiments) (PDF)

## Notes

This research study has received approval from the relevant ethical review board/institutional review board (IRB) at Hospital de Clnicas de Porto Alegre under protocol number #0075-2020. The study was conducted following the ethical principles outlined in the Declaration of Helsinki.
