# Supplementary material for: Bioactive Composite Membranes: Preclinical Analysis of PBAT/BAGNb Composition to Enhance the Quality of Guided Bone Regeneration
Source: ACS Appl Mater Interfaces. 2025 Feb 12;17(8):11774–81. doi: 10.1021/acsami.4c20580 (PMC11873951; doi:10.1021/acsami.4c20580)
Supplement: Supplementary file 1 — am4c20580_si_001.pdf [file am4c20580_si_001.pdf]

Supporting Information

Bioactive Composite Membranes: Pre-clinical Analysis of PBAT/BAGNb Composition to Enhance the Quality of Guided Bone Regeneration

Gabriela de Souza Balbinot [a], Vicente Castelo Branco Leitune [b], Rosane Michele Duarte Soares [c], Fernanda Visioli [d], Deise Ponzoni [e], Fabricio Mezzomo Collares [f*]

[a] DDS, MSc, Assistant Professor - Dental Materials Laboratory, School of Dentistry. Universidade Federal do Rio Grande do Sul, 90035-003, Porto Alegre, RS, Brazil. gabriela.balbinot@ufrgs.br ORCID: 0000-0001-9076-2460

[b] DDS, MSc, PhD, Adjunct Professor - Dental Materials Laboratory, School of Dentistry. Universidade Federal do Rio Grande do Sul, 90035-003, Porto Alegre, RS, Brazil. vicente.leitune@ufrgs.br ORCID: 0000-0002-5415-1731

[c] MSc, PhD, Associate Professor Polymeric Biomaterials Laboratory (Poli-BIO), Institute of Chemistry, Universidade Federal do Rio Grande do Sul, 91501-970, Porto Alegre, RS, Brazil. soaresr@ufrgs.br ORCID: 0000-0002-5225-7559

[d] DDS, MSc, PhD, Adjunct Professor - Patology Laboratory, School of Dentistry. Universidade Federal do Rio Grande do Sul, 90035-003, Porto Alegre, RS, Brazil. fernanda.visioli@ufrgs.br ORCID: 0000-0002-4033-8431

[e] DDS, MSc, Ph.D. Chair Professor, Oral and Maxillofacial Surgery Unit Universidade Federal do Rio Grande do Sul 90035-003, Porto Alegre, RS, Brazil. deponzoni@yahoo.com.br ORCID: 0000-0003-2855-7495

[f] DDS, MSc, PhD, Adjunct Professor - Dental Materials Laboratory, School of Dentistry. Universidade Federal do Rio Grande do Sul, 90035-003, Porto Alegre, RS, Brazil Porto Alegre, RS, Brazil. fabricio.collares@ufrgs. ORCID: 0000-0002-1382-0150

*fabricio.collares@ufrgs.br

Figure S1. ARRIVE checklist with the guidelines for reporting animal studies.

ARRIVE

The ARRIVE guidelines 2.0: author checklist

The ARRIVE Essential 10

These items are the basic minimum to include in a manuscript. Without this information, readers and reviewers cannot assess the reliability of the findings.

Item		Recommendation	Section/line number, or reason for not reporting
Study design	1	For each experiment, provide brief details of study design including:	2.2 Study design
		a. The groups being compared, including control groups. If no control group has been used, the rationale should be stated.	
		b. The experimental unit (e.g. a single animal, litter, or cage of animals).	2.5 Statistical analysis
Sample size	2	a. Specify the exact number of experimental units allocated to each group, and the total number in each experiment. Also indicate the total number of animals used.	2.2 Study design
		b. Explain how the sample size was decided. Provide details of any *a priori* sample size calculation, if done.	2.5 Statistical analysis
Inclusion and exclusion criteria	3	a. Describe any criteria used for including and excluding animals (or experimental units) during the experiment, and data points during the analysis. Specify if these criteria were established *a priori*. If no criteria were set, state this explicitly.	2.2 Study design
		b. For each experimental group, report any animals, experimental units or data points not included in the analysis and explain why. If there were no exclusions, state so.	Not applicable
		c. For each analysis, report the exact value of *n* in each experimental group.	2.4 X-ray computed microtomography
Randomisation	4	a. State whether randomisation was used to allocate experimental units to control and treatment groups. If done, provide the method used to generate the randomisation sequence.	2.2 Study design
		b. Describe the strategy used to minimise potential confounders such as the order of treatments and measurements, or animal/cage location. If confounders were not controlled, state this explicitly.	2.2 Study design
Blinding	5	Describe who was aware of the group allocation at the different stages of the experiment (during the allocation, the conduct of the experiment, the outcome assessment, and the data analysis).	2.2 Study design
Outcome measures	6	a. Clearly define all outcome measures assessed (e.g. cell death, molecular markers, or behavioural changes).	2.4 X-ray computed microtomography
		b. For hypothesis-testing studies, specify the primary outcome measure, i.e. the outcome measure that was used to determine the sample size.	2.4 X-ray computed microtomography
Statistical methods	7	a. Provide details of the statistical methods used for each analysis, including software used.	2.5 Statistical analysis
		b. Describe any methods used to assess whether the data met the assumptions of the statistical approach, and what was done if the assumptions were not met.	
Experimental animals	8	a. Provide species-appropriate details of the animals used, including species, strain and substrain, sex, age or developmental stage, and, if relevant, weight.	2.2 Study design
		b. Provide further relevant information on the provenance of animals, health/immune status, genetic modification status, genotype, and any previous procedures.	
Experimental procedures	9	For each experimental group, including controls, describe the procedures in enough detail to allow others to replicate them, including:	2.3 Surgical procedures
		a. What was done, how it was done and what was used.	
		b. When and how often.	
		c. Where (including detail of any acclimatisation periods).	
		d. Why (provide rationale for procedures).	
Results	10	For each experiment conducted, including independent replications, report:	3. Results
		a. Summary/descriptive statistics for each experimental group, with a measure of variability where applicable (e.g. mean and SD, or median and range).	
		b. If applicable, the effect size with a confidence interval.	
